# Research advances in the construction of stem cell-derived ovarian organoids

**DOI:** 10.1186/s13287-024-04122-3

**Published:** 2024-12-31

**Authors:** Tianyue Zhang, Mengtong Zhang, Sichen Zhang, Shaowei Wang

**Affiliations:** 1 Department of Gynecology and Obstetrics, Beijing Hospital, National Center of Gerontology, Institute of Geriatric Medicine, Chinese Academy of Medical Sciences, Beijing, 100730 People’s Republic of China; 2 Beijing Hospital, National Center of Gerontology, Institute of Geriatric Medicine, Chinese Academy of Medical Sciences and Peking Union Medical College, Beijing, People’s Republic of China

**Keywords:** Ovarian organoid, Extracellular matrix, Synthetic matrices, Decellularization, Pluripotent stem cells

## Abstract

Ovarian organoids are essential in female reproductive medicine, enhancing our understanding of ovarian diseases and improving treatments, which benefits women’s health. Constructing ovarian organoids involves two main processes: differentiating induced pluripotent stem cells (iPSCs) into germ and ovarian somatic cells to restore ovarian function and using extracellular matrix (ECM) to create a suitable ovarian microenvironment and scaffold. Although the technology is still in its early stages, future advancements will likely involve integrating high-throughput analysis, 3D-printed scaffolds, and efficient iPSC induction, driving progress in reproductive and regenerative medicine.

## Background

Organoid technology, which employs three dimensional(3D) culture systems and matrix scaffolds to regulate cellular signals and induce stem cells to form organ-like structures, is a crucial supporting research on disease mechanisms, drug screening, and personalized precision medicine. It has been applied to studies of various organs, including those of the colon, prostate, breast, pancreas and ovaries [1, 2].

The ovaries regulates follicle development and the secretion of steroid hormones. Ovarian dysfunction can impact both quality of life and fertility, with hormonal changes leading to issues such as infertility, osteoporosis, cardiovascular disease, and depression.It was reported that 1.0-1.5% of women under the age of 40 experience menopause due to premature ovarian failure (POF), and one in five to six couples suffer from infertility [3]. Ovarian-related diseases, includind POF, polycystic ovary syndrome (PCOS), autoimmune disorders, and iatrogenic conditions caused by chemotherapy, radiotherapy, and oophorectomy, can disrupt hormonal dynamics and follicular maturation, leading to premature menopause and reduced fertility [4]. Therefore, understanding how to maintain and to restore of ovarian function is crucial for preserving fertility and enhancing quality of life with aging.

Clinically, hormonal therapies are the primary approach for restoring ovarian function. However, their efficacy is limited, and they may result in side effects such as ovarian hyperstimulation syndrome and deep vein thrombosis [5]. Ovarian organoids, with their reproductive and endocrine functions, mimic the ovarian microenvironment and support follicle maturation, offering a promising therapeutic strategy for fertility preservation in POF patients.

Numerous studies have summarized advancements in patient-derived organoids (PODs) within the field of gynecological oncology [1, 2, 6]. Ovarian cancer organoids, due to their high heterogeneity and ability to preserve the diversity of the original tissue, have significantly enhanced our understanding of ovarian cancer pathogenesis and drug screening, thereby advancing personalized and precision medicine. However, replicating all ovarian cell types is challenging due to the ovary’s mix of germ and somatic cells. Recent advancements in developmental biology and stem cell research have enabled the differentiation of stem cells into germ-like cells and gametes, paving the way for 3D-induced ovarian organoids through tissue engineering. Despite this progress, methods for inducing ovarian cell types from stem cells and optimizing the extracellular matrix (ECM) remain underexplored. This article reviews recent developments in stem cell differentiation and ECM engineering, aiming to guide the construction of ovarian organoids for in vitro oocyte formation and in vivo hormonal therapy, thereby advancing reproductive medicine.

## Inducing stem cells to form germ cells

Germ cells are fundamental to human reproduction. Follicle growth occurs in two stages. The initial slow phase lasts several weeks in small rodents and several months in large mammals, characterized by the proliferation of granulosa cells and an increase in the diameter of both the follicle and the oocyte. In the second stage, the follicles respond to follicle-stimulating hormone (FSH) and luteinizing hormone (LH), leading to follicular cavity formation, steroid hormone synthesis, and the pre-ovulatory phase [7].

In modern infertility treatments, improving the quality and quantity of oocytes is crucial. In particular, there is a need to develop new technologies to produce human gametes, improve oocyte quality and quantity, and integrate these methods into assisted reproduction [8].

### Inducing stem cells to form primordial germ cells

Stem cell therapy is a cornerstone of regenerative medicine. Based on the mechanisms underlying oocyte development into gametes, research focuses on developing methods to induce the maturation of secondary oocytes from stem cells that are capable of fertilization. In mice, stem cell development occurs in two stages: spontaneous and induced differentiation. Initially, researchers obtained primordial germ cells (PGCs) by allowing stem cells to differentiate spontaneously. In 2003, Hübner et al. [9] discovered that mouse embryonic stem cells (mESCs) cultured in heated inactivated serum could develop into oocytes, undergoing meiosis and forming blastocyst-like structures. Toyooka and colleagues [10] later used the testis capsule to generate sperm from mESCs in a medium containing lymphocyte inhibitory factor (LIF), although they did not assess the sperm’s reproductive function. In 2004, Clark et al. [11] studied the spontaneous differentiation of human embryonic stem cells (hESCs) into embryoid bodies (EBs) that expressed germ cell-specific markers. However, the efficiency of these spontaneous differentiation methods for generating PGCs from stem cells was quite low, ranging from only 0.5–3.6% per EB.

To improve induction efficiency, research has increasingly focused on inducing PGCs from induced pluripotent stem cells (iPSCs). In 2008, Ying et al. [12] advocated for culturing pluripotent stem cells (PSCs) in a combination of MAPK, GSK, and LIF (known as 2i) to maintain germline-competent naive PSCs in what they referred to as the “ground state.” Building on this, several induction methods have been proposed. In 2009, Ohinata et al. [13] used BMP4, LIF, SCF, BMP8b, and EGF to induce germ cell characteristics in mouse epiblast cells, achieving an induction success rate of up to 45%. In 2011, Hayashi et al. [14] reported the successful derivation of PGCs from mESCs for the first time. This technique was used in conjunction with co-culturing gonadal cells (testicular mesothelial cells/ovarian somatic cells) to reconstruct mouse ovaries and testes, ultimately producing offspring. However, due to chromosomal abnormalities after meiosis, the offspring birth rate was low [15]. In 2014, Kimura et al. [16] found that inhibiting the ERK signaling pathway using MEK inhibitors mesodermal differentiation system while promoting germ cell differentiation in the mesodermal differentiation system of OP9 feeder cells. Although functional sperm could not be produced, this finding offered new insights for inducing iPSCs. Mouse PGC induction serves as a model to validate hypotheses on stem cell induction while adhering to ethical restrictions on human research, laying the foundation for human-induced PSCs (hiPSCs).

Regarding human germ cell development, Chuang et al. [17] demonstrated that hESCs could spontaneously differentiate into germ cell-like cells in a differentiation medium. Similar to methods used in animal stem cells, the synergistic action of BMP4 and WNT3A promoted the differentiation of hESCs into germ-like cells, achieving a differentiation rate of 31–47%. A significant advancement came when Irie et al. [18] established defined culture conditions to standardize the derivation of human primordial germ cell-like cells (hPGCLCs) from hESCs and hiPSCs. They maintained near-ground-state pluripotency by pre-culturing PSCs in a medium containing four inhibitors: CHIR99021 (a GSK-3 inhibitor), PD0325901 (a MEK inhibitor), SB203580 (a p38 MAPK inhibitor), and SP600125 (a JNK inhibitor). To induce hPGCLCs, they cultured the PSCs in this medium for two days with bFGF/TGFβ, then transitioned to a suspension culture containing BMP2 (or BMP4), LIF, SCF, and EGF. The derived hPGCLCs exhibited gene expression and epigenetic patterns similar to human PGCs (hPGCs). Subsequent studies introduced various induction processes with different factors. However, due to ethical constraints regarding human subjects, the reproductive function of these induced cells could not be verified.

**Fig. 1:**
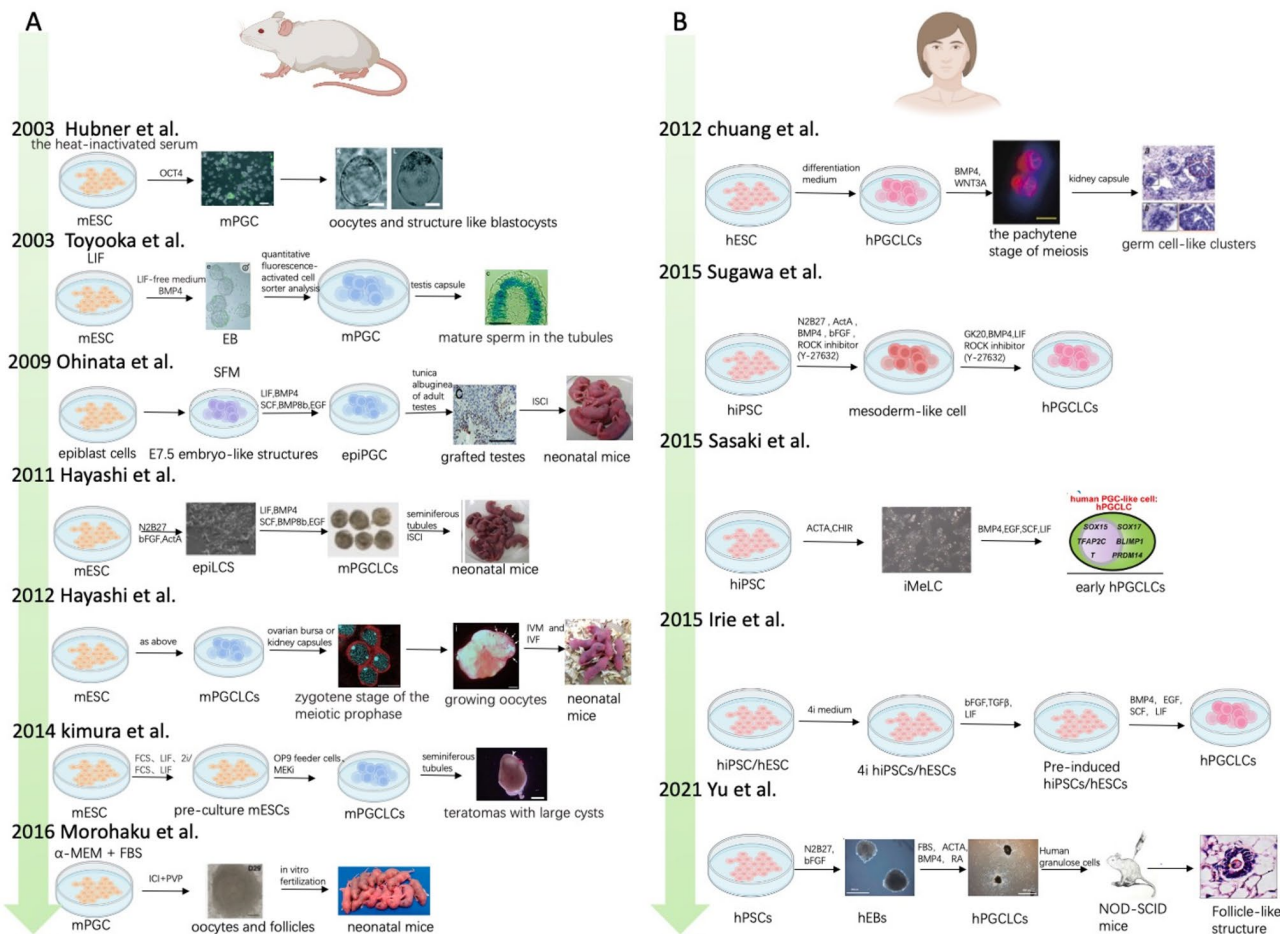
Process of inducing iPSCs into PGCLCs (**A.** Mouse induction process; **B.** Human induction process)

In 2015, Sasaki et al. [19] applied hiPSCs, initially using the GSK3 inhibitor (CHIR99021) and Activin A to derive induced mesoderm-like cells (iMeLCs). These cells were then converted into BVSC-positive hPGCLCs using Irie’s method. In 2021, researchers from National Taiwan University reported the induction of early germ cells from hESC and hiPSCs using RA, BMP4, and Activin A. When these interacted with human granulosa cells and were cultivated in vivo in mice, they developed into follicle-like cells [8] ( Fig. 1).

Human gamete development is a complex process involving numerous transcription factors that regulate differentiation. Various combinations of transcription factors are currently used to induce human stem cells into PGCs, with induction efficiencies ranging from 27–47%—below the ideal level. Future research should focus on the epigenetics and developmental biology of human gamete differentiation, identifying key transcription factors for stem cell differentiation into gametes and optimizing their combinations to improve induction efficiency. Due to the uniqueness of the human population, reproductive function validation has not yet been achieved. However, with the advancement of genomic research, future studies may be able to verify genetic stability through omics approaches, paving the way for potential clinical applications.

### Primordial germ cells differentiating into gametes

To generate mature follicles, ongoing research focuses on establishing in vitro conditions for inducing PGCs into more advanced germ cells. Initially, researchers co-cultured PGCs with ovarian somatic cells or testicular supporting cells to achieve oocyte maturation and meiosis. For example, in 2016, researchers reported the use of Transwell-COL culture to co-culture mESCs and female gonadal somatic cells to form mouse ovaries, which then induced functional gamete production [20]. In 2018, Yamashiro et al. [21] co-cultured in vitro induced hPSCs with mouse ovarian somatic cells for 120 days. This process resulted in the gradual development of oocyte-like cells entering prophase of meiosis, although their survival rate was less than 10%. In 2022, Yang et al. [22] used fetal ovarian somatic cells from miscarried fetuses (7–8 weeks old) and hPGCs/oogonia derived from hiPSCs to create allogeneic recombinant ovaries (IrOvaries). These ovaries were reconstructed under ovarian capsules in severe combined immune deficiency( SCID) mice, generating haploid human oocytes in vivo, with a 3.2% transformation rate from mitosis to meiosis. However, the low induction efficiency, long induction times, and ethical concerns regarding the use of allogeneic fetal ovaries, along with the complexity and high costs of the technique, severely limit their potential for clinical translation. Therefore, efficient and simplified induction methods are urgently needed.

Researchers aim to identify specific induction and transcription factors that drive early germ cells into meiosis. Some studies have highlighted factors promoting germ cell differentiation into meiosis. In 2011, Eguizabal et al. [23] used retinoic acid (RA) to pre-induce primordial germ cell-like cells (PGCLCs) from human iPSCs. Subsequently, they applied human LIF (hLIF), forskolin (FRSK), bFGF, and a CYP26 inhibitor (R115866) to induce meiosis, producing haploid-like cells with an efficiency of 0.4–2.3%. This demonstrated that transcription factors could induce meiosis, although the resulting cells resembled sperm-like structures. In 2012, researchers found that overexpressing VASA and/or DAZL in hESCs and iPSCs increased meiosis-related marker expression, promoting meiotic progression in vitro [24]. Chuang et al. [17] discovered that BMP4 and Wnt3A could further induce hESC differentiation into germ cells reaching the pachytene stage of meiosis, which were successfully implanted into kidney capsules. However, their reproductive functionality was not validated.

In 2017, Jung’s team [25] used DAZL and BOULE to induce hESCs, regulating the exit from pluripotency and entry into meiosis. They further applied recombinant human GDF9 and BMP15, which induced these meiotic germ cells to form follicle-like cells (FLCs). In 2020, Abdyyev et al. [26] used BMP4 and recombinant human Activin A (rhActA) to culture hiPSCs and hESCs into late-stage germ cells, followed by RA induction. Although RNA-seq analysis revealed changes in meiosis-related genes, haploid cells were not successfully generated. These results indicate that current in vitro conditions are insufficient to complete meiosis.

Identifying optimal conditions for this process is crucial for ovarian organoid development, as producing mature gametes remains a key goal. Disorders in oocyte development are among the primary causes of infertility. In vitro gametogenesis can enable genetic screening, treat infertility, and improve reproductive health. Future research should focus on elucidating germ cell differentiation mechanisms both in vivo and in vitro, identifying factors beyond RA that promote haploid formation, and ensuring genetic and epigenetic stability. These challenges remain central to future research in this field.

### Regulatory network of hPGC development

**Fig. 2:**
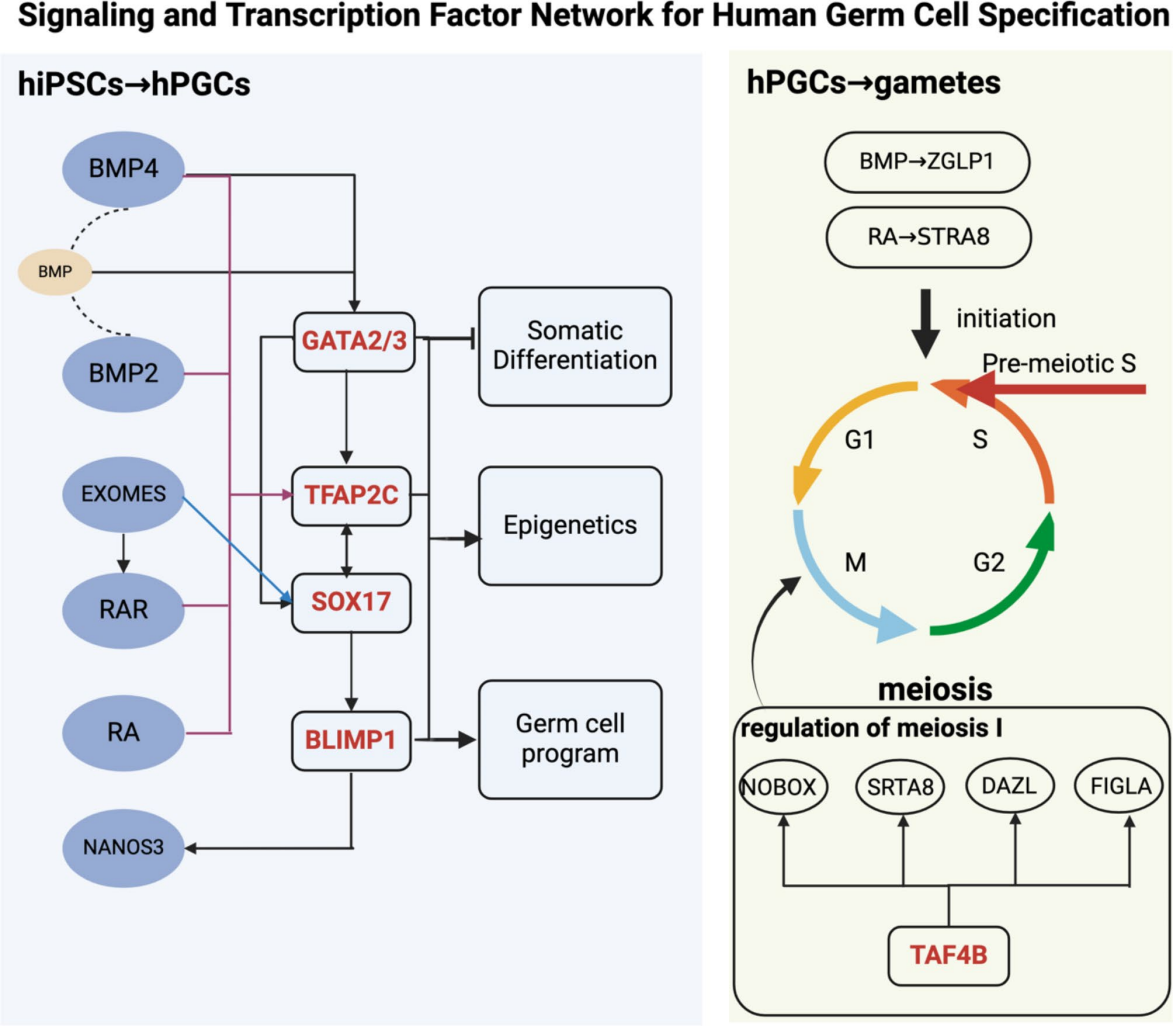
Signaling and transcription factor network for human germ cell development (divided into two regulatory processes involving signals and transcription factors: induction of hiPSCs into hPGCs and completion of meiosis by hPGCs. Arrows indicate stimulation or upregulation, while lines indicate inhibition.) (Created with BioRender.com)

Understanding the signaling pathways and transcription factor networks governing human germ cell specification is crucial for efficient female gamete production. Identifying and inducing key upstream factors can activate germ cell development genes, leading to the efficient production of the desired female gametes. Current studies suggest that GATA2 or GATA3, SOX17, and TFAP2C are critical transcription factors in the process of inducing hPGCs from hiPSCs. These factors suppress somatic differentiation while promoting epigenetic reprogramming and germ cell development (Fig. 2) [27, 28].

In the induction of female gametes from hPGCs, transcription factor TAF4b is vital for meiosis I and the establishment of a healthy primordial follicle reserve by regulating key genes, including Stra8, Figla, Nobox, and Dazl [29]. The BMP-ZGLP1 axis and RA-STRA8 axis guide female germ cells into meiosis, while the STRA8-RB axis facilitates S-phase progression during meiosis, significantly contributing to female germ cell differentiation (Fig. 2) [30]. However, the expression, regulation, and interactions of downstream genes involved in meiosis remain poorly understood. Further exploration of these processes is crucial not only for understanding fetal oocyte development but also for gaining insights into oocyte aging, both of which are key to achieving successful in vitro differentiation.

Future research should prioritize constructing a more comprehensive signaling network for hPGC development, focusing on the intricate interactions between pathways and their specific contributions to germ cell fate determination. This approach will help identify precise signaling factors, ultimately improving the efficiency and accuracy of in vitro gametogenesis. These advancements will enable infertility treatments and support the development of reproductive medicine and regenerative biology.

### Animal models for gamete development

Most research on in vitro gametogenesis (IVG) relies on mouse models. Human germ cells and mouse PGCs exhibit similar expression profiles for many key genes, including OCT4, NANOG, BLIMP1, TFAP2C, LIN28, SSEA1, cKIT, NANOS3, DAZL, and VASA [31]. However, gametogenesis and developmental mechanisms differ between species. For example, the key regulator Sox17 in hPGCs is expressed only transiently in mouse PGCs (mPGCs), whereas SOX15 expression is closely associated with hPGCs. Another difference lies in the expression of Sox2, which is downregulated in hPGCs but restored in mPGCs [32]. The activity of Blimp1 also varies: It suppresses mesodermal programs in mPGCs but inhibits neuronal development programs in hPGCs.

Despite these differences, mouse models remain valuable for genetic studies and provide significant reference value for researching human gametogenesis. Other animal models, such as rats, pigs, cows, and goats, can also be used to induce PGCLCs from iPSCs [32, 33]. Among these, pigs have shown particularly promising results. Porcine iPSCs, first induced to EpiLCs with Activin A and bFGF and then differentiated into PGCLCs, are treated with BMP4, BMP8B, SCF, hLIF, and EGF, similar to the mouse model for PGCLC induction. Notably, porcine PGCLCs exhibit SOX17 regulatory trajectories similar to those of humans [34].

Research on gametogenesis induced from mammalian stem cells provides valuable insights for human stem cell studies. Animals like pigs, with developmental biology similarities with humans, help validate regulatory factors and ensure genetic stability. Meanwhile, small mammals, being cost-effective and easier to handle, play an important role in advancing reproductive research. Selecting suitable animal models based on species traits is critical for advancing stem cell meiosis induction. Ultimately, this research aims to develop methods for generating gametes from hiPSCs in vitro. Studying induction processes in different animals will help establish a foundation for identifying appropriate stem cell sources, organoid carriers, and optimized methodologies [12].

### Stem cell-induced ovarian somatic cells

The formation of ovarian organoids requires the participation of both germ cells and ovarian somatic cells, making it crucial to generate ovarian somatic cell-like cells in vitro. Previous studies have used ovarian somatic cells derived from germ cell-depleted sources, which pose challenges such as genetic instability and immune rejection during clinical translation. Novel techniques for generating fetal ovarian somatic cell-like cells from ESCs/iPSCs could eliminate the need for embryonic somatic cells. Yoshino et al. [35, 36] used several signaling factors (WNT, BMP, SHH, and RA) to stimulate signaling pathways guiding the differentiation of mouse pluripotent cells into E12.5 ovarian somatic cell-like cells. When co-cultured with mPGCLCs, these FOSLCs form reconstituted ovarian-like compounds (rOvarioids) capable of producing functional oocytes.

In 2021, Wang et al. [37] found that treating cells with a combination of AM580 and vitamin C induced the expression of Foxl2 and Gata4, activating RA- and Wnt-related pathways to produce E12.5 gonadal somatic cell-like cells (E12.5 GSCLCs). Co-culturing these cells with PGCs activated meiosis in vitro, although with much lower efficiency than that achieved with E12.5 GSCs.

Producing ovarian somatic cells is a relatively challenging process because these cells constitute a complex group, including ovarian surface epithelium (OSE) cells, granulosa cells, and theca cells. Some studies have reported successful induction of certain types of OSE cells. In 2013, Liu et al. [38] reported that inducing microRNA-17-3P expression while inhibiting vimentin expression transformed hiPSCs into estrogen-sensitive ovarian epithelial-like cells (OSE-like cells). These cells were transplanted into a mouse model of POF, where they secreted estradiol.

Only a few studies have successfully generated granulosa cells in vitro. In a 12-day multi-step protocol, Lan et al. [39, 40] used recombinant signaling proteins to induce hESCs to differentiate into functional ovarian granulosa-like cells, although yields were low (12–36%). In 2016, Liu et al. [41] utilized various cell growth factors (TGF-β and human growth hormone) and hormones (E2, AMH, inhibin α, and inhibin β) at different time points to induce iPSCs to generate granulosa-like cells after 12 days. These cells were validated in a mouse model of POF, where they repaired ovarian damage and improved ovarian function. Similarly, Lipskind et al. [33] found that EBs derived from mESCs and human amniocyte-derived induced PSCs (hAdiPSCs) could serve as biologically relevant models for ovarian granulosa cell differentiation and steroid cell production, although conversion efficiency was relatively low. In 2023, Smela et al. [39] demonstrated that overexpressing transcription factors NR5A1 and RUNX1 or RUNX2 was sufficient to produce granulosa-like cells, with an induction efficiency of up to 70%, and tested their endocrine functions. There are no reports of generating theca cells from PSCs, with current reports limited to theca cells derived from theca stem cells [42].

While the induction of ovarian somatic cells from stem cells still requires optimization, recent progress in understanding granulosa cell development has led to advancements in restoring ovarian function in endocrine disorders. However, human-like pulsatile hormone secretion has not yet been replicated, and high induction rates and full-type differentiation of ovarian somatic cells have yet to be achieved. Future research efforts should focus on replacing exogenous gonadal cells with defined synthetic factors or iPSC-derived gonadal cells to pave the way for constructing ovarian organoids.

Current research on human ovarian somatic cell development focuses mainly on transcriptomics, highlighting stage-specific gene expression. Reported key transcription factors such as FOXL2, GATA4/6, NR5A1, WT1, and SOX9 are important for this process but require further investigation [43]. This is partly due to the limited progress in inducing human ovarian somatic cells, making it difficult to validate their roles in development.The interaction between follicular and somatic cells is crucial, influenced by hormones like FSH and LH, as well as gene interactions. Transcriptomic studies suggest that the NOTCH signaling pathway regulates oocyte-mediated granulosa cell proliferation and differentiation [44]. Further research is needed to identify key regulatory factors and understand hormonal regulation, laying the groundwork for in vitro induction of ovarian somatic cells.

In conclusion, recent studies have demonstrated the induction of ovarian germ cells, granulosa cells, and epithelial cells from hiPSCs. However, ethical concerns prevent full verification of their genetic and epigenetic stability, raising concerns about potential mutations and germ cell tumors. The simultaneous induction of multiple ovarian cell types remains a challenge, and meiosis in the absence of ovarian somatic cells has yet to be achieved. Further research is needed to develop functional artificial ovaries with endocrine and reproductive capabilities to treat female infertility and improve reproductive health.

### Extracellular matrix construction

The construction of organoids requires stem cell induction and a suitable ECM for mechanical and nutritional support in 3D culture. The ECM is a vital network that supports stem cell growth, structural integrity, and the exchange of essential substances between organoids and culture medium. It regulates cell behavior and maintains tissue-specific functions, making ECM selection critical for successful organoid construction [6, 45]. ECM characteristics have been extensively leveraged in tissue engineering and regenerative medicine research, aiming to restore the function of damaged or dysfunctional tissues. The ovarian ECM environment is both complex and dynamic, regulated by periodic changes in endocrine factors from both systemic circulation and local signals. Its properties enhance interactions between follicles and somatic cells, making it a key focus in regenerative reproductive medicine [46].

Matrigel, derived from mouse Engelbreth–Holm–Swarm (EHS) sarcoma, is the most commonly used ECM for organoid construction. It supports cell growth with essential proteins and growth factors and has been widely utilized in stem cell differentiation and tumor organoid studies. However, Matrigel’s tumor origin, batch variability, instability, immunogenicity, limited tunability, and lack of scalability limit its use in advancing organoid culture technologie [47].

To address these limitations, two innovative approaches have been proposed. The first involves using biomaterials with well-controlled components and mechanical properties, while the other employs tissue decellularization techniques to create tissue-specific matrices [1].

Biomaterials, with their defined biochemical composition, mechanical properties, and low cost, are expected to become an alternative to Matrigel. Dadashzadeh et al. [48] reviewed the literature on various 3D culture systems designed to support follicle development. Natural polymers such as alginate, tyramine-based HA hydrogel, collagen, fibrin, alginate–collagen mixtures, and plasma clots have been employed to establish these systems. These approaches have enhanced follicle integrity, diameter, and oocyte maturation. Synthetic hydrogels, such as polyethylene glycol (PEG) derivatives, have also been used to mimic natural ovarian ECM. However, recent research remains limited to the follicle stage, and further studies are required to evaluate their potential for early stem cell attachment and gamete induction. Significant challenges, including reproducibility, hypoxia, insufficient nutrient supply, and immune rejection, must be addressed before clinical application [7].

To enhance follicle survival, researchers have proposed adding angiogenic factors like VEGF, bFGF, and EGF to scaffolds, which can promote vascularization by attracting endothelial cells. Limited studies have confirmed that VEGF and bFGF can induce angiogenesis and improve follicle survival [48–51]. Optimizing the mechanical and biological properties of synthetic materials, combined with appropriate regulatory factors, could create a microenvironment conducive to vascularization and cell growth, potentially enabling synthetic biomaterials to replace Matrigel for efficient culture and industrial-scale production.

Decellularized ECM (dECM) is another promising alternative, as it removes cellular components—primarily DNA—from tissues while preserving the tissue’s biochemical and structural properties. With low immunogenicity and a non-tumor origin, dECM is ideal for organoid construction, providing scaffolds with optimal porosity, stiffness, and elasticity for tissue repair. Methods for decellularizing ovarian tissues and their application in ovarian research are summarized in Table 1. The combination of dECM and hydrogels has also shown promise. Studies indicate that ovarian cells grown in recombinant dECM and alginate hydrogels exhibit high biocompatibility. Notably, depleting elastin microfibril interface-1 (EMILIN1) from synthetic polymers combined with decellularized mouse ovarian tissue has been shown to support follicle survival in vitro [52]. Nikniaz and colleagues [53] corroborated these findings, reporting an 85.9% viability rate for transferred follicles within the ECM-alginate scaffold after seven days.

Decellularized ovarian scaffold hydrogels provide an ideal platform for supporting follicle development and restoring ovarian function, offering both physical and chemical support for oocyte maturation. Due to ethical limitations, obtaining dECM from human ovaries is challenging. Therefore, the most feasible approach involves using dECM from other species for human applications. Current research on ovarian dECM hydrogels is restricted to in vitro studies focused on promoting follicle growth and oocyte maturation, with no in vivo studies available. Further exploration of suitable hydrogel materials and their characteristics is necessary to complete the development of ovarian organoids [54, 55].

**Table 1:** Studies related to ovarian decellularization

Tissue	Method	Species	Effect in vitro	Effect in vivo	Ref
Small tissue pieces	Rotated in 0.1% SDS at room temperature for 24 h	Bovine and human	Supports ovarian somatic cell growth	Initiates puberty in ovariectomized mice	[56]
1.5-mm-thick pieces	1% Triton X-100 for 9 h and 0.5% SDS for 3 h at room temperature with agitation (100 rpm); treated with 200 U/mL DNase I in PBS at 37 °C for 12 h	Bama miniature pigs	Non-cytotoxic for rat ovarian cells, supported rat granulosa cell penetration and estradiol (E2) secretion	Minimal immunogenic response in rats	[57]
Manageable strips	1% sodium lauryl ester sulfate (SLES) for 48 h, followed by DNase I in PBS for 24 h	Women	Suitable cytocompatibility of the scaffolds	Oocyte growth, folliculogenesis, and endocrine function (estradiol and progesterone) in rats	[58]
Tissue pieces	0.1% sodium dodecyl sulfate (SDS) for 18–24 h, followed by 24 h of 1 mg/mL DNase treatment and washing	Women	Supports survival of human follicles and growth of murine follicles, with 39% reaching antral stages	Follicular recovery rates after three-weeks grafting were low but similar for both human (25%) and murine follicles (21%)	[59]
Small pieces	0.5% SDS for 3 h, 1% Triton X-100 for 9 h, and 2% deoxycholate for 12 h	Gilts	Encouraged rapid cell adhesion and migration, with repopulating cells increasing in number and aggregating into cluster-like structures	-	[60]
Whole organ	Immersed in 0.5% sodium dodecyl sulfate (SDS) in deionized water for 3 h and incubated overnight in 1% Triton X-100 in deionized water	Porcine	porcine ovarian cells(pOCs) recellularize the ECM-based scaffold and survive for 7 days, verifying ECM-based scaffolds’ ability to properly drive and address cell differentiation	-	[61]
Pieces	Immersed and shaken in a combined solution of 2% sodium deoxycholate and 4% Triton X-100 for 36 h, with an exchange every 6 h. Followed by 36 h in 1% sodium dodecyl sulfate. Tissues were incubated in RNase/DNase solution (80 U/mL) for 6 h at 37 °C	Porcine	Scaffolds support GC survival for at least 15 days but might not sustain follicle growth for 7 days	Mainly caused an innate immune response in mice	[62]
Small pieces	Treated in a 0.69% tris (hydroxymethyl) aminomethane and 2.5 mM sodium deoxycholate solution at 50 rpm for 24 h	Porcine	Preantral follicles cultured in a biomimetic ovary, and embryos were generated from MII oocytes	-	[63]
Slices	Treated with 0.1% sodium dodecyl sulfate (SDS) in 1× phosphate-buffered saline (PBS)	Bovine	Supports follicle growth and survival	-	[52]
Slices	Treated with 0.1% SDS and 0.02 M NaOH for 12 h	Bovine	No toxicity to human ovarian cells when cocultured for up to 72 h	-	[64]
Whole ovary	Submerged in liquid nitrogen for 10 h, treated with 0.5% SDS for 2 h, 1% Triton X-100 for 8 h, and 2% deoxycholic acid for 8 h. Rinsed with DI-H2O for 6 h, changing every 2 h to remove cell remnants	Porcine	Higher number of granulosa cells in vitro co-culture	-	[65]

Products derived from dECM from other organs, such as cartilage, adipose tissue, and muscle, including decellularized tissue sheets, have already entered clinical practice. However, the application of dECM for ovarian tissue remains in its early stages. One significant challenge is the recellularization process, which requires complex co-culture systems to reseed all necessary cell types to reconstruct the ovarian microstructure. Furthermore, maintaining an artificial organ in culture demands substantial nutritional and oxygen support, and extensive research is needed to overcome these limitations [66].

3D printing has introduced innovative methods for creating ovarian-like organ ECMs, although ovarian 3D printing is still in its infancy. In 2017, Laronda et al. [67] attempted to generate prosthetic ovaries using 3D-printed microporous scaffolds made from gelatin. They reported partial restoration of ovarian functions, including hormone secretion and follicle production, in sterilized mice, culminating in the birth of healthy offspring. More recently, in 2022, T Wu et al. [4] designed and fabricated a 3D artificial ovary using a gelatin-methacryloyl (GelMA) bio-ink extrusion method. In the same year, Zheng et al. [68] employed 3D printing to create dECM scaffolds from pig ovaries, which were co-cultured with mouse ovarian fragments to construct artificial ovaries. In vivo and ex vivo experiments with mice confirmed the significant role of these constructs in repairing damaged ovaries. Given its excellent biomimetic capabilities, 3D printing is expected to play a critical role in developing supportive ovarian components in future research.

In summary, while Matrigel remains essential for research, bio-synthetic materials offer unmatched advantages in tissue engineering and regenerative medicine due to their controllability and scalability. For ovarian tissue engineering, biomaterials must balance biological factors (e.g., cell attachment and differentiation) with tissue-like mechanical properties to support organoid formation and improve transplantation outcomes. Optimizing material type, concentration, and stiffness is crucial for preserving and reconstructing the ovarian environment. Combining 3D printing with dECM technology holds great promise for constructing ovarian organoids.

### Current status of ovarian organoids

The ovary is a critical organ for maintaining female reproductive and endocrine functions. Current fertility preservation strategies include cryopreservation of embryos, immature or mature oocytes, and ovarian tissue cryopreservation and xenotransplantation. Hormone replacement therapy (HRT) is often used to maintain endocrine function. However, these approaches have notable limitations, including economic burden and increase health risks. For example, infertility patients may be unable to produce viable embryos, while HRT increases the risk of ovarian cancer and is unsuitable for patients with estrogen-dependent cancers or a history of deep vein thrombosis [69]. Additionally, transplanted ovarian tissue lasts only 4–5 years and may provoke strong immune responses [57]. To address these challenges, ovarian organoids are emerging as a promising alternative to restore ovarian function without the burden of the original disease [64].

**Fig. 3:**
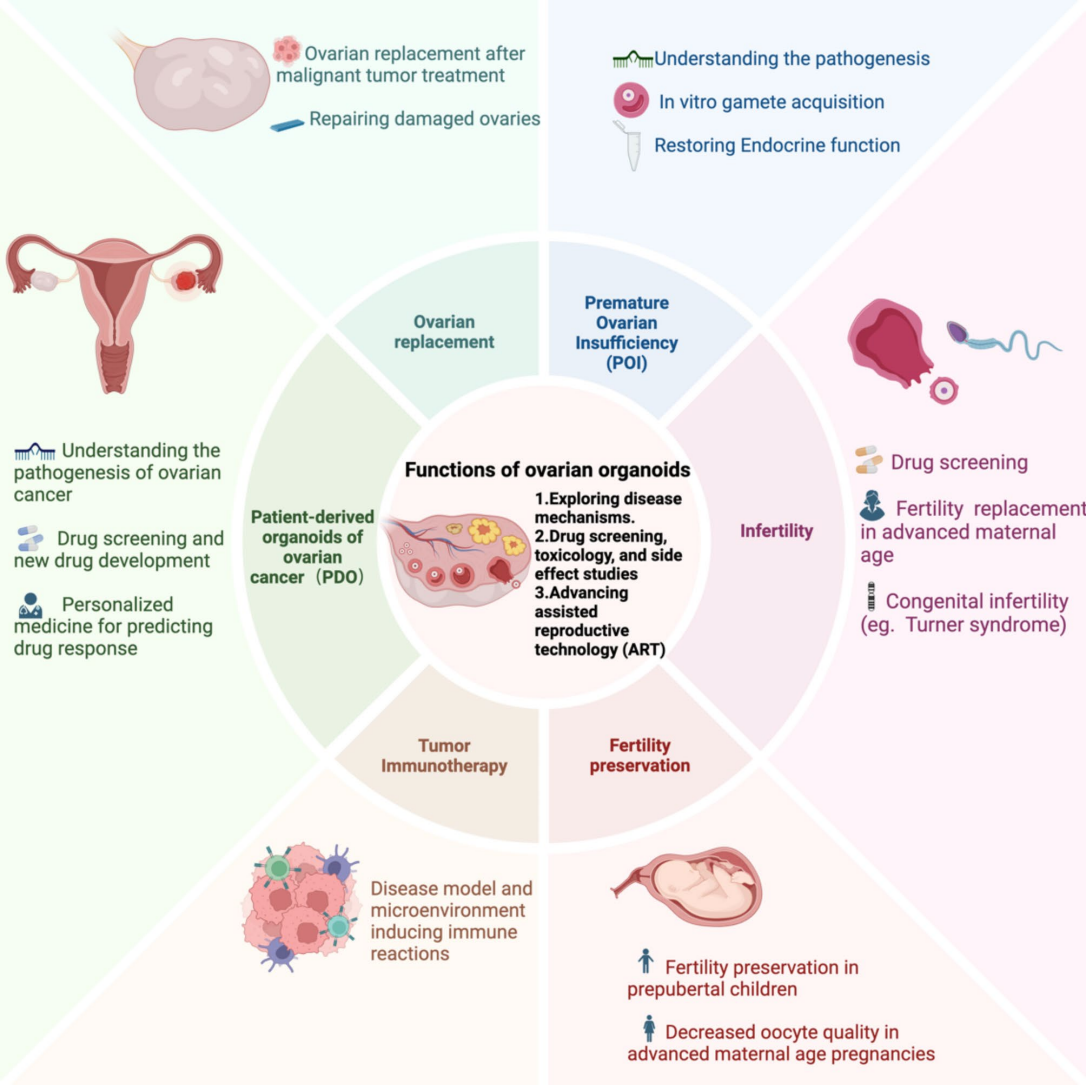
Functions of ovarian organoidsz(Ovarian organoids hold potential for exploring disease mechanisms and treatments, drug screening [e.g., for ovarian insufficiency, endometriosis, and ovarian cancer], as well as infertility treatment and fertility preservation.) (Created with BioRender.com)

Rapid advancements in developmental biology and cellular regulation have shed light on the developmental mechanisms of germ cells in different species, enabling the directed differentiation of iPSCs to construct ovarian organoids. One of the latest trends in bioengineering, organoid development involves the 3D arrangement and culture of cells to replicate tissue and organ functions in vitro. Ovarian organoids have diverse applications, such as PODs for studying disease mechanisms, drug screening (e.g., for ovarian insufficiency, endometriosis, ovarian cancer), and immunotherapy. They can also replace ovarian endocrine function, induce the production of mature oocytes in vitro for infertility treatment, and preserve fertility, offering vast potential for clinical applications (Fig. 3).

Ovarian organoids are remarkably promising tools in reproductive medicine. Their ability to be monitored and manipulated in a controlled environment makes them ideal models for investigating the mechanisms underlying ovarian-related diseases. In 2021, Li et al. [70] became the first to use female germline stem cells (FGSCs) co-cultivated with Matrigel to generate organoid models. They found that mature oocytes derived from these organoids could produce offspring and exhibit endocrine functions similar to those of natural ovaries. These organoids were subsequently used to screen for the cytotoxicity of salinomycin. In 2023, Ma and colleagues [71] demonstrated through in vitro experiments that MitoQ, due to its antioxidant properties, reduces oxidative stress in follicles, thereby protecting oocytes and granulosa cells, improving oocyte quality, and enhancing follicle development. Researchers from Harvard University combined hiPSC-induced ovarian somatic cells with hPGCLCs, aggregating these two cell types in low-binding U-bottom wells, followed by transfer to air–liquid interface Transwell cultures. This resulted in the first reported fully human ovarian organoids. Despite challenges such as cell flattening, the inability to provide functional theca cells, and a shorter survival time, this marked a significant advance in ovarian organoid research [39].

However, researchers have not yet succeeded in inducing the formation of all ovarian cell types from hiPSCs in vitro with full endocrine and reproductive functionality. Consequently, creating clinically applicable ovarian organoids remains a distant goal, necessitating further research and improvements. A major challenge is maintaining an adequate nutritional and oxygen supply for these structures before and after transplantation. Addressing biocompatibility issues is also crucial for transitioning from laboratory studies to clinical use [66].

Before applying such technologies in medical practice, safety concerns must be resolved. These include genomic and epigenomic instability in PSC-derived gametes, which may pose health risks to patients and their offspring. Therefore, evaluating the quality and integrity of these gametes is essential. Ethical and social issues must also be carefully considered. While deriving germ cells from PSCs could benefit individuals with congenital infertility, the potential underutilization of germ cells from hiPSCs could lead to societal challenges, such as defining parental identity. Thus, it is crucial to establish strict standards, ethical guidelines, and regulations that align with societal norms as this technology develops [3, 72].

## Conclusion, challenges, and future perspectives

The construction of ovarian organoids involves inducing PSCs and creating an ECM. While PSCs can generate ovarian germ cells, granulosa cells, and epithelial cells, the induction of theca cells and meiosis is still not possible. Further research into human development and epigenetics is needed to identify key factors for co-induction, which is vital for creating functional ovarian organoids. Additionally, stimulating in vivo ovarian hormone secretion in vitro is necessary to produce mature and genetically stable follicles. Although Matrigel is commonly used in research, its limitations—stemming from being derived from mouse tumors—highlight the need for ECMs with more appropriate mechanical and biological properties. The ultimate goal is to develop an ECM that supports cell and follicle growth, is stable, and can be mass produced.

**Fig. 4:**
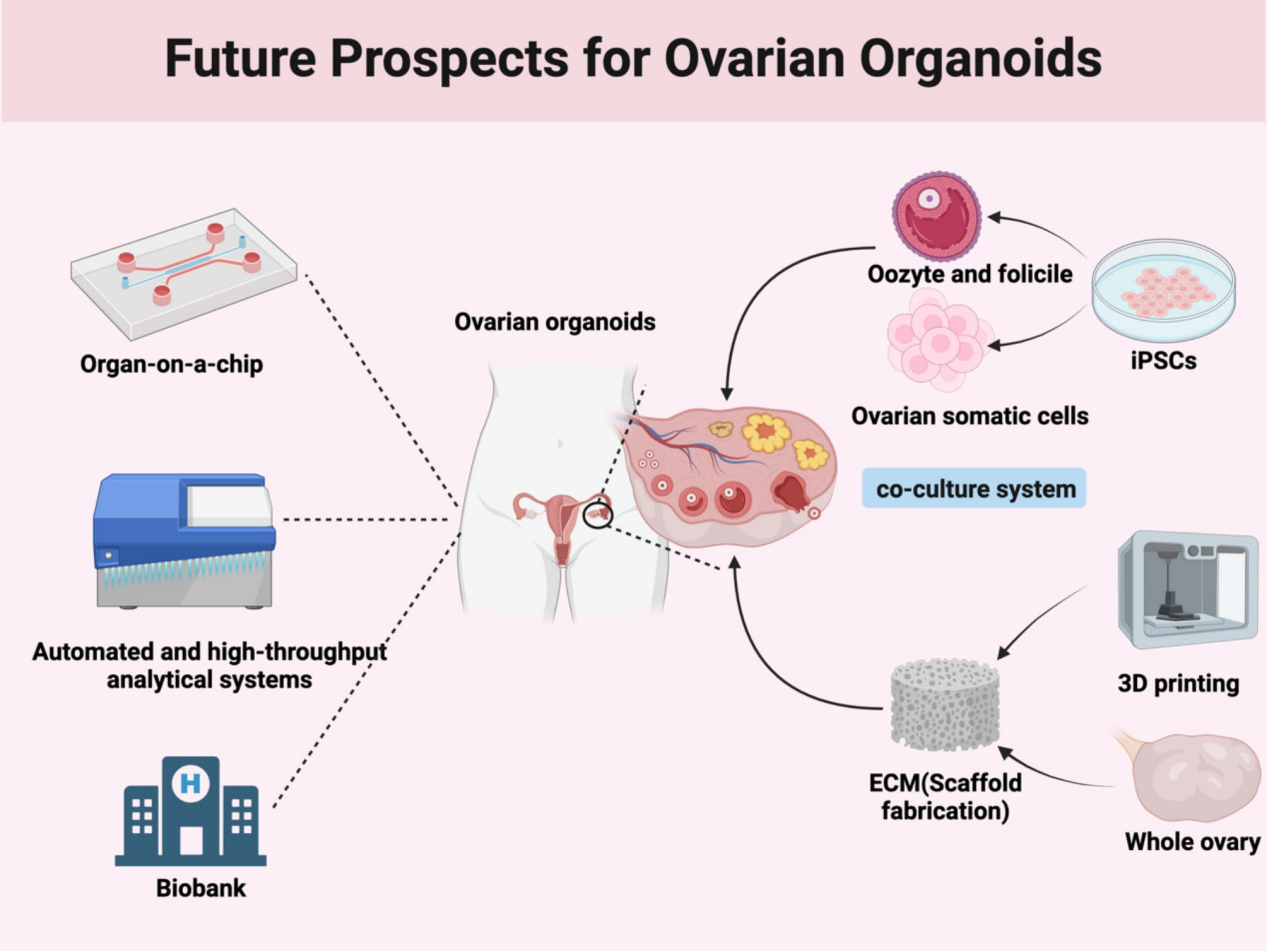
Future prospects for ovarian organoids (Inducing iPSCs with key transcription factors and high-performance ECM, combined with automated high-throughput analytical technologies, organoid-on-a-chip platforms, and biobanks, represents the future of organoid research.) (Created with BioRender.com)

Future advances in gene engineering technologies such as CRISPR-Cas9 and 3D printing are expected to drive progress in organoid technology [73, 74], resulting in safer iPSCs and improved pregnancy outcomes for women with genetic conditions. This technology holds promise for personalized biological repair of ovarian dysfunction or insufficiency. As society increasingly emphasizes women’s reproductive health, the demand for ovarian organoids is expected to increase. Future developments will focus on organ-on-a-chip platforms, automation, high-throughput systems, and organoid biorepositories, enabling large-scale production to support disease research and drug screening (Fig. 4).

Human ovarian organoids hold immense potential in reproductive medicine. The generation of various ovarian cell types from iPSCs and the development of ovarian ECM are advancing rapidly. However, significant challenges remain. With continued research efforts and strong societal demand, ovarian organoids are expected to ethically and effectively support patients with ovarian-related diseases. They promise to enable treatments for infertility, restoration of ovarian function, and fertility preservation, heralding a new era of personalized reproductive and regenerative medicine.

## Data Availability

No data was used for the research described in the article.

## Abbreviations

3D: Three dimensional
POF: Premature ovarian failure
PCOS: Polycystic ovary syndrome
PODs: Patient-derived organoids
FSH: Follicle-stimulating hormone
LH: Luteinizing hormone
mESCs: Mouse embryonic stem cells
LIF: Lymphocyte inhibitory factor
hESCs: Human embryonic stem cells
EBs: Embryoid bodies
PGCs: Primordial germ cells
PSCs: Pluripotent stem cells
hPGCLCs: Human Primordial Germ Cell-Like Cells
hiPSCs: Human induced pluripotent stem cells
hPGCs: Human primordial germ cells
hAdiPSCs: Human amniocyte-derived induced pluripotent stem cells
iMeLCs: Induced Mesoderm-like Cells
RA: Retinoic acid
PGCLCs: Primordial germ cell-like cells
ESCs: Embryonic stem cells
iPSCs: Induced pluripotent stem cells
FLCs: Follicle-like cells
IVG: In vitro gametogenesis
mPGCs: Mouse primordial germ cells
OSE: Ovarian surface epithelium
dECM: Decellularized extracellular matrix
HRT: Hormone replacement therapy
HG-SOC: High-grade serous ovarian cancer
